# Delayed interval delivery of the second twin in a woman with altered markers of inflammation

**DOI:** 10.1186/s12884-018-1848-4

**Published:** 2018-06-04

**Authors:** George Daskalakis, Panagiotis Fotinopoulos, Vasilios Pergialiotis, Mariana Theodora, Panagiotis Antsaklis, Michail Sindos, Nikolaos Papantoniou, Dimitrios Loutradis

**Affiliations:** 1 1st Department of Obstetrics and Gynecology, Athens Medical School , Alexandra General Hospital, 9 Aristeidou Street , 17563 P. Faliro, Athens, Greece; 2 3rd Department of Obstetrics and Gynecology, Athens Medical School, Attikon General Hospital, Athens, Greece

**Keywords:** Intertwin, Delayed, Delivery, Monitoring, Inflammation

## Abstract

**Background:**

Delayed interval intertwin delivery rates are expected to rise during the next years as potent and targeted tocolytic agents are employed and antenatal surveillance methods become more sophisticated and specific in predicting the critical delivery timepoint of optimal perinatal outcome.

**Case presentation:**

We present a case of delayed intertwin delivery after delivery of the first twin due to premature prelabor rupture of the membranes. Maternal serum White Blood Cells and C-Reactive Protein levels remained high until delivery of the second twin (34 days after the first was delivered), although maternal temperature remained constant. The mother underwent close antenatal surveillance and she was hospitalized. She had an uncomplicated delivery of the second twin at 29^+ 2^ weeks by cesarean section due to an abnormal Non-Stress Test.

**Conclusion:**

We strongly suggest future evaluation of maternal serum inflammatory markers among these rare cases as these could predict intraamniotic infection.

## Background

The rate of multiple pregnancies over the last three decades has increased dramatically due to extended implementation of Artificial Reproduction Techniques (ART) [1]. The rate of twin pregnancies in the U.S. has climbed to 33.2 per 1.000 births in 2009 (increased by 76% since 1980) [2] . Higher order pregnancies remained constant from 2001 until a steep rise of 4% (153.5 per 100,000 births) in 2009 [2]. About 50% of live births in twins are preterm (< 37 weeks of gestational age), whereas higher order pregnancies are almost entirely born premature preterm. Furthermore it seems that ART twin pregnancies (regardless as to whether they are In Vitro Fertilization or not) have a tendency to be born prematurely than the naturally conceived ones [3] .

Although preterm deliveries seem to rise the last 20years, an unexpected inverse variance with birth weight is also noted, leading to the assumption that fetal growth is improved, possibly due to better antenatal surveillance [4]. Perinatal outcome is negatively affected by low and very low birth weight. Therefore, all efforts by means of improved antenatal surveillance and therapeutic tocolysis should be focused on controlling prolongation of the pregnancy. Twin pregnancy that is complicated by very preterm delivery of the first fetus is a challenge in modern obstetrics and, to date, evidence regarding the optimal time of delivery of the second twin is lacking. Although, clinical chorioamnionitis (including fever, uterine tenderness and presence of contractions) is an absolute indication for delivery, to date, there is no consensus regarding the method of antenatal surveillance of twin pregnancies that are complicated with expulsion of the first fetus in the second, or early third trimester. In the present study we report a case of delayed interval delivery of the second twin 34 days following delivery of the first.

## Case presentation

A thirty four years-old G2P1 woman was admitted in the high risk pregnancy unit of our The First Dpt of Obstetrics and Gynecology at 23^+ 4^ weeks of twin gestation due to premature prelabor rupture of the membranes (*PPROM*). The twins were dichorionic according to the first trimester ultrasound scan. The patient reported the presence of increased “vaginal discharge” during the last week. Nitrazine tape test was positive showing amniotic fluid leakage. She had an uncomplicated previous singleton term vaginal delivery 3 years ago. Her personal medical history revealed the presence of hypothyroidism that was treated with thyroxine 175 mcg twice a day, an appendicectomy 12 years ago and cervical cryotherapy for Human Papilloma Virus (HPV) 1 year ago.

During physical examination, she was normotensive, afebrile and the cervix was not dilated or effaced. Laboratory examinations at admission were obtained and revealed the presence of mild leucocytosis (12,300/μl) and an elevated C- Reactive Protein (CRP) (40.98 mg/L with upper normal laboratory limit of 1.0 mg/L), while both dipstick urine examination and urinary cultures were normal. The patient received amoxicillin and metronidazole regimen eight hourly for 10 days and betamethasone 12 mg intramuscularly with a repeated dose at 24 h. Ultrasound examination revealed the presence of two embryos with positive cardiac function that weighted 535 and 606 g. The first of them had an amniotic fluid index (*AFI*) of 5 cm and the second an AFI of 14 cm. The patient’s cervical length was 28 mm and funneling was not noted. Ultrasonographic and laboratory assessment was performed every 3 days. Three days later WBCs were raised (14,200/μl) whereas CRP value declined at 2.90 mg/L.

One week following admission (24^+ 4^) the patient experienced blood stained brownish vaginal secretions and the vaginal examination revealed a Bishop score of 8. She was transferred to the labor ward where she delivered a female that weighted 550 g. Manual extraction of the placenta failed and the umbilical cord was ligated just above the level of the external cervical os. The vagina was rinsed with antiseptic solution (povidone iodine, Betadine®) and she remained in the labor ward under close surveillance of vital signs and fetal heart rate for the next 4 h. No signs of active labor were noticed. After informed consent and detailed counselling about the possible benefits and complications, the woman opted for delayed delivery of the second twin.

**Fig. 1:**
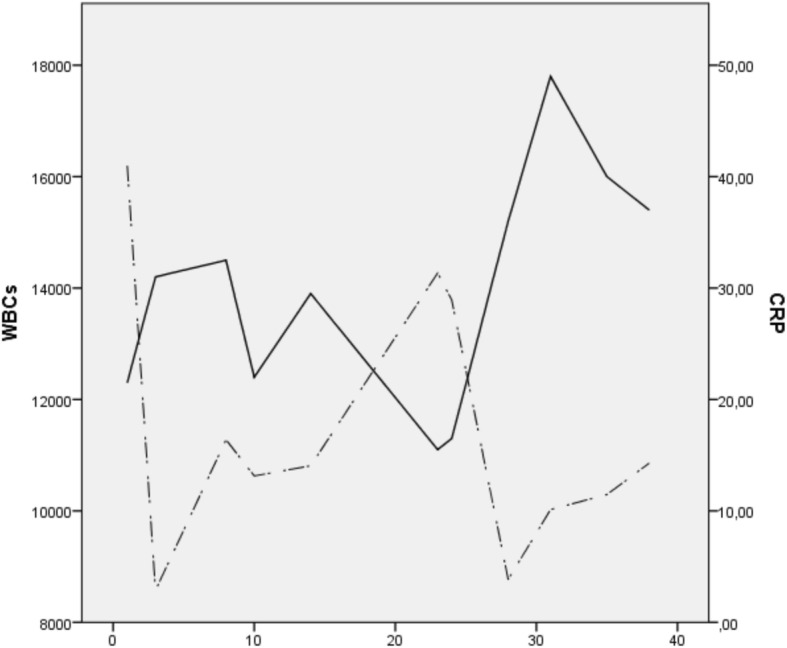
Time-trends in WBC (cells/ mm^3^, solid line) and CRP (mg/L, dotted line) values during hospitalization• Delayed interval delivery of the second twin represents a clinical challenge.• Subclinical chorioamnionitis cannot be excluded and its impact on the pregnancy course and perinatal outcome remains unknown• WBCs and CRP may provide evidence of deterioration of the patients` pregnancy course• Future studies are needed to investigate the predictive accuracy of these indices.

The next day the delivered twin died from respiratory distress syndrome. Blood samples were obtained from the patient that once again revealed raised white blood cells (WBCs) (12,400/μl) and increased CRP (13.14 mg/L). The patient remained in the high-risk pregnancy department and 5 days later she had a new blood and urine examination along with urine cultures that revealed elevated WBCs (13.900/μl), an a steep rise in CRP (31.78 mg/L) along with the presence of enterobacteriae spp. An expert in infectious diseases was advised and the patient received cefuroxime 750 mg eight hourly for 7 days. The surveillance protocol involved close laboratory assessment (WBC and CRP levels three times a week), ultrasonographic evaluation twice a week, vital signs clinical assessment (arterial pressure, heart rate, temperature) and electronic fetal monitoring (non stress test, NST) twice a day . The fluctuations of CRP and WBC values during the patient’s hospitalization are shown in the Fig. 1. She remained afebrile with no clinical evidence of chorioamnionitis. A repeated dose of steroids was administered to the patient during her 26th and 27th day of hospitalization (28th week of gestation).

The second female fetus was finally delivered 34 days after the first fetus (29^+ 2^) with cesarean section due to an abnormal NST. The neonate weighed 1150 g and had an Apgar score of 7 at the first minute and 9 at 5 minutes**.** It remained in the Neonatal Intensive Care Unit (NICU) for about 4 weeks.

## Discussion

Prematurity and very low birth weight pose great risks for the neonate due to inability of its organs to adapt. Respiratory Distress Syndrome (RDS), sepsis, necrotizing enterocolitis, intraventricular hemorrhage and periventricular leucomalacia) are common complications that are attributed to prematurity. In a previous study, tocolytics, antenatal steroids and surfactant administration within the first 2 hours following delivery were the most important predictors of neonatal survival for twins born between 22 and 26 weeks of gestational age [5]. The advancement of gestational age is very important in extremely premature neonates (less than 27 weeks of gestational age) as each day improves survival rates and decrease the duration of hospitalization in the NICU [6] . In singleton, neonatal survival following delivery at 24, 25, and 26 weeks is estimated to be 31.2, 59.1, and 75.3%, respectively [7]. In multiple gestations, the mortality rate reaches 32% from 23 to 25 weeks’ gestational age, compared to 19.2% from 26 to 27 weeks’ gestational age and 11.1% in all gestational age [8].

In multiple gestations with preterm delivery of the first baby, the second is usually delivered within a short time frame. Occasionally, however, uterine contractions stop and the cervix reconstitutes.The condition is referred to as Delayed Interval Delivery (DID) of the second twin. Due to the rarity of DID, standard protocols for the management of these patients don’t exist. Contraindication for delayed delivery are fetal distress, congenital abnormalities, preterm rupture of membranes of the remaining fetus, chorioamnionitis, monoamniotic or monochorionic pregnancies, and severe vaginal blood loss. The aseptic ligation of the umbilical cord stump close to the placenta reduces the risk of developing infection due to maceration and is practiced in these cases. Some authors routinely perform cervical cerclage immediately after the first delivery while others don’t recommend it. Arabin and van Eyck (2009) suggest that cerclage should be best avoided due to concerns of chorioamnionitis. On the contrary, Zhang et al. (2003) support cerclage, because it can minimize fetal membranes’ exposure to vaginal bacteria and acidity, prolonging delay interval.

Numerous studies were published during the last decade addressing the case of delayed interval delivery of the second twin. One of the largest series was recorded from a population based study in the U.S. [9] .The authors concluded that when fetal expulsion of the first twin occurred between 22 and 23 weeks the prolongation of the intertwin interval could decrease perinatal mortality of the second twin. Interestingly, however, this beneficial effect persisted up to 3 weeks following delivery of the first twin. When the interval was prolonged more than 4 weeks the incidence of a Small for Gestational Age (SGA) second twin was also increased. Although 5 min Apgar scores less than 7 significantly decreased, they were not accompanied by a subsequent reduction in rates of respiratory distress syndrome. Arabin et al. confirmed these results stating that although the prolongation of the interval led to decreased perinatal mortality and morbidity of the second twin it was followed by an increase in the prevalence of SGA neonates [10]. The same authors extended their study in triplet gestations and found that regardless the interval between the first and second fetus, the third triplet was delivered at a maximum interval of 2 days, following delivery of the second triplet.

Farkouh et al. observed in pregnancies with delivery of the first twin during the 22th week of gestation that the concurrent placement of cerclage increased the intertwin delivery interval compared to pregnancies with delivery of the first twin after removal of an elective cerclage (≥49 vs ≤26 days) [11] . The same authors concluded that even modest intervals of delivery could improve neonatal morbidity and mortality. However, we must underline the fact that all women in their series received routine antibiotic and tocolytic therapy and amniocentesis of the remaining twin was offered prior to cerclage placement to preclude intraamniotic infection. Furthermore, all pregnant women were informed that although the procedure could increase the intertwin delivery interval, the fetal outcome and the occurrence of maternal morbidity could not be excluded. Zhang et al. retrospectively analyzed 7 cases that were offered cervical cerclage after delivery of the first twin and found that the procedure did not increase the risk for intrauterine infection [12]. Recent systematic review by Feys et al., shows clear evidence of lower mortality risk of the second twin with DID [13] .

In our case, the leading twin after PPROM and amniotic fluid leakage was finally delivered by normal labor. We chose not to perform an episiotomy during its delivery. Maternal laboratory examinations were initially suggestive of the presence of infection, however there was no clinical evidence, as uterine tenderness and fever were absent. The mother was informed regarding the potential existence of chorioamnionitis and was offered the chance to perform amniocentesis to preclude infection; however she declined the operation and wished to continue her pregnancy with conservative treatment. WBCs and CRP were routinely assessed every 3 days, or once a day when a steep rise was observed (Fig. 1).

WBCs fluctuated between higher normal limits (12,000/μl) and 24,700/μl, whereas CRP was continuously at least 2-fold higher than higher normal laboratory limit (although the fluctuation of its levels could not be clinically interpreted). During delivery the vagina was cleansed with antiseptic solution and a high umbilical cord ligation was performed (above the level of the external cervical os). Given the presence of biochemical signs of infection we chose to avoid cervical cerclage and tocolytic therapy. The patient remained hospitalized. In a retrospective evaluation of 73 women with PPROM investigators found that maternal CRP levels were not effective in predicting chorioamnionitis [14]. However, an increased CRP could potentially predict future chorioamnionitis development. In another study, maternal CRP levels of more than 20 mg/L were found predictive of funisitis among singleton pregnancies [15]. In their systematic review Van de Laar et al. conclude that although CRP was found to be a moderate predictor of chorioamnionitis, its use isn’t yet supported [16]. Popowski et al. noted that maternal WBC count has a poor predictive value, and is only considered highly specific when the threshold of 16,000/μl is exceeded [17]. Park et al. suggested that the evaluation of maternal WBC and CRP levels, along with parity and gestational age could be used as a predictive model of intraamniotic infection in singletons with PPROM [18]. The area under the curve of the model was particularly high (0.848 (95% CI 0.788–0.908) and its sensitivity and specificity reached values of 81 and 75% respectively. Our case differs from the aforementioned studies, as the latter are focused in singleton pregnancies, therefore not taking into account the potential confounders that may lead to extrauterine infection after the live birth of the first twin.

## Conclusions

Delayed interval intertwin delivery rates are expected to increase during the next years as antenatal surveillance becomes more intensive and new tocolytic agents become more popular. Elective cerclage may be considered in twin pregnancies with delivery of the first twin before 23 weeks of gestation. However, this approach should be taken into account in women with no signs of infection. Although we strongly suggest routine performance of amniocentesis for the evaluation of the amniotic fluid for inflammatory markers and routine cultures, a number of patients may still deny this invasive technique. Close antenatal surveillance of both mother and fetus is strongly suggested among these special cases. Given the fact that subclinical chorioamnionitis cannot be precluded, and its effects on the pregnancy course remain undetermined in cases of delayed interval delivery of the second twin, laboratory assessment of markers of inflammation could be potentially considered in these special cases. White blood cells WBCs and CRP have been widely adopted to trace down infections in internal medicine and to follow the course of infectious diseases. Their actual predictive value in delayed delivery of the second twin remains uninvestigated. Our case report presents such a case and may be used as a reference for future studies in this field. These should specifically investigate the sensitivity and specificity of blood biomarkers in predicting chorioamnionitis and fetal infection to help reduce perinatal maternal and neonatal morbidity and mortality.

## Abbreviations

*AFI*: Amniotic fluid index
ART: Artificial reproduction techniques
CRP: C- reactive protein
DID: Delayed interval delivery
NICU: Neonatal intensive care unit
NST: Non stress test
*PPROM*: Premature prelabor rupture of the membranes
RDS: Respiratory distress syndrome
SGA: Small for gestational age
WBC: White blood cells
